# Assessment of the Knowledge of Healthcare Professionals Regarding Radiological Examination in Lactating Mothers: A Cross-Sectional Survey Study in the Kingdom of Bahrain

**DOI:** 10.7759/cureus.76070

**Published:** 2024-12-20

**Authors:** Bedoor Alomran, Latifa Fakhroo, Maryam Al Khalifa, Fatima Alhakim, Ghada A Hassan, Eman Shajira

**Affiliations:** 1 Radiology, Bahrain Defence Force Royal Medical Services, Riffa, BHR; 2 Diagnostic Radiology, Bahrain Defence Force Royal Medical Services, Riffa, BHR; 3 College of Medicine, Royal College of Surgeons in Ireland - Bahrain, Busaiteen, BHR; 4 Pediatrics, Bahrain Defence Force Royal Medical Services, Riffa, BHR; 5 Pediatric Nursing, Bahrain Defence Force Royal Medical Services, Riffa, BHR; 6 Pediatrics and Neonatology, Bahrain Defence Force Royal Medical Services, Riffa, BHR

**Keywords:** breastfeeding practice, healthcare professionals, lactating women, radiation, radiological imaging, x-ray exposure

## Abstract

Objectives

Breastfeeding is critical for a mother's health, as well as the development and survival of her infant. Healthcare personnel are a reliable source of information for breastfeeding mothers during and after radiological procedures, assuming their understanding is appropriate. As a result, this study analyzed healthcare professionals' knowledge of breastfeeding and radiological tests on lactating mothers.

Methods

A cross-sectional survey was implemented using a validated questionnaire containing 25 items about breastfeeding habits and radiation imaging including male and female healthcare staff in primary and tertiary hospitals in Bahrain. The survey comprised 384 responders who were doctors, nurses, radiology technicians, and other allied health professionals.

Results

In general, 284 (74%) participants scored outstanding knowledge on questions about the health benefits of breastfeeding for both mothers and newborns. However, there was a clear lack of awareness concerning the use of imaging modalities and contrasts on lactating mothers, with only 117 (30.5%) participants scoring an "Excellent" knowledge score. Factors associated with basic knowledge included breastfeeding practice, source of knowledge, and years of experience. The availability of CT/MRI/nuclear scans was one factor related to radiological imaging knowledge. Participants' age, academic background, and profession had strong correlations with fundamental and radiological knowledge.

Conclusion

Overall, health professionals' awareness and understanding of breastfeeding was average; there is a lack of knowledge of radiological imaging on lactating mothers, which may lead to the discontinuation of breastfeeding following radiological examination. Our study advises incorporating the impacts of exposure to radiological imaging into breastfeeding education programs to increase healthcare providers' knowledge and thereby encourage breastfeeding practice.

## Introduction

Breastfeeding constitutes one of the most efficacious methodologies to promote the health and survival of infants, as breastmilk furnishes all the requisite energy and nutrients essential for the initial six months of life. Furthermore, breastmilk enhances the infant's immune system and has the potential to provide protection against non-communicable diseases in later life, such as diabetes and obesity. However, World Health Organization (WHO) data indicate that less than 50% of infants under six months of age are exclusively breastfed, as recommended by WHO and the United Nations Children's Fund (UNICEF) [1].

The Global Strategy for Infant and Young Child Feeding jointly developed by the UNICEF and WHO articulates that the development of infants represents a paramount public health concern, and it is recommended that all infants undergo exclusive breastfeeding for the initial six months, continuing to receive breast milk until two years of age to complement the introduction of other nutritional foods [2]. The act of breastfeeding confers numerous health advantages for both mothers and their infants. It significantly diminishes the likelihood of breast and ovarian cancer occurrence among women who engage in breastfeeding practices [3].

Over the last decade, there has been a rise in the utilization of radiologic examinations for pregnant and lactating women as a supplementary tool in diagnostic assessments. These imaging techniques should be employed judiciously and only when alternative methods are unavailable to address a pertinent clinical inquiry or to offer medical advantages to the patient [4]. Healthcare professionals often encounter challenges when it comes to exposing breastfeeding women to radiation imaging, primarily due to insufficient understanding of the associated health risks [5]. This situation can create unwarranted anxiety for patients, healthcare providers, and the broader community, which may lead to the unnecessary avoidance of beneficial diagnostic procedures, delays in diagnosis, or even the premature cessation of breastfeeding [5,6].

Numerous radiopharmaceuticals are eliminated via breast milk, necessitating adherence to guidelines concerning the interruption of breastfeeding to ensure that the infant's effective dose remains under 1 millisievert (mSv) [6]. A minimal fraction of iodinated contrast agents or gallium-based contrast materials is transferred into breast milk and subsequently absorbed by the infant [4,7]. For scheduled contrast-enhanced examinations, lactating mothers may utilize a breast pump before the radiation exposure to store milk for future use [8].

The appropriate application of imaging techniques and the use of contrast materials in lactating mothers requires an understanding of their safety. Several strategies can be implemented to reduce radiation exposure, such as employing radionuclides with short half-lives and administering only the minimum necessary dose of radiopharmaceutical agents, particularly during extended imaging procedures.

Enhancing the knowledge and practices within healthcare is the most effective approach to increasing both the prevalence and duration of breastfeeding [9]. Healthcare professionals are pivotal in providing education to breastfeeding mothers; however, this requires them to receive adequate training and education, including instruction on various radiological techniques and the associated risks for both mothers and infants. Consequently, it is vital for all healthcare personnel to be well-educated and trained in order to apply best-practice breastfeeding policies that promote breastfeeding and enhance health outcomes [10].

There are numerous studies evaluating healthcare professionals' understanding of breastfeeding in the literature; however, we did not encounter any study focused on their knowledge of radiological investigations in lactating mothers. Consequently, this study was designed to evaluate healthcare professionals' awareness of the advantages of breastfeeding and the application of radiological investigations. The findings of this study will contribute to the promotion of breastfeeding practices in Bahrain.

## Materials and methods

This was a prospective, internet-based, cross-sectional study conducted in Bahrain from August 2022 to October 2022. Participants included nurses, medical doctors, radiology technicians, and various allied healthcare professionals across Bahrain. Participation was voluntary, ensuring that all responses remained confidential and anonymous. The study protocol was reviewed and approved by the Research Ethical Committee of Bahrain Defence Force Royal Medical Services (approval number: 2022-675).

Study tool and data collection

The questionnaire was designed after a thorough review of existing literature [11]. Additional questions regarding radiological knowledge were created by the researchers, validated by expert panels, and pre-tested on a small group from the target population. The survey was organized into four distinct sections. The initial section focused on demographic information. The second and third sections included a total of 25 questions related to breastfeeding, with the first section containing 13 questions aimed at assessing general knowledge about breastfeeding, while the second section featured 12 questions specifically addressing radiological knowledge. Each question was accompanied by two columns for respondents to indicate whether the statement was true or false. The fourth and final section comprised a single question regarding the source of the respondents' knowledge. The survey was disseminated to healthcare workers throughout Bahrain, encompassing both private and public sectors, via a link to the survey.

Scoring system

We employed a scaled scoring system to evaluate knowledge levels. Each correct answer selected by a participant earned one point, and no points were subtracted for incorrect responses. The scores for basic and radiological imaging knowledge were determined based on the number of correctly answered questions in each section. The total score for Basic Knowledge was calculated out of 13, with classifications as follows: a score of 5 or lower was considered Poor, scores between 6 and 9 were deemed Average, and scores from 10 to 13 were rated as Excellent. For the Radiological Knowledge score, calculated out of 12, a score ranging from 0 to 4 was classified as Poor, 5 to 8 as Average, and 9 to 12 as Excellent. Finally, the Overall score, which totaled 25, categorized scores of 13 or lower as Poor, scores from 14 to 19 as Average, and scores between 20 and 25 as Excellent.

Sample size estimation

With an estimated population size of 3500, the initial sample size (n₀) for a proportion was calculated with the following formula: $n₀ =\frac{(Z&sup2; \times p \times (1 - p))}{E&sup2;}$. Here, Z represents the Z-score (for instance, 1.96 corresponds to a 95% confidence level), p is the estimated proportion (0.5 is used if unknown), and E denotes the margin of error (0.05 in this case). 

Calculating this gives: $n₀ =\frac{(1.96&sup2; \times 0.5 \times (1 - 0.5))}{E&sup2;} = 384$.

Since the population size is finite (N = 3500), we apply the finite population correction using the formula $&eta;=\frac{_{&eta;_{0}}}{1+\frac{&eta;_{0}-1}{N}}$. This results in $&eta;=\frac{384}{1+\frac{384-1}{3500}}$ ≈ 347. Therefore, the necessary sample size for a finite population of 3500 was approximately 347.

Statistical analysis

Continuous variables were expressed as median values along with their interquartile ranges (IQR), while categorical variables were summarized using frequencies and percentages. To compare the median total scores based on respondents' characteristics, either the Mann-Whitney U test or the Kruskal Wallis test was employed, depending on the data characteristics. All analyses were performed using IBM SPSS Statistics for Windows, Version 26.0 (Released 2019; IBM Corp., Armonk, New York, United States). A p-value of less than 0.05 was deemed statistically significant.

## Results

Demographic data

The research sample included 384 individuals, with 346 (90.1%) identifying as female and 38 (9.9%) as male. The participants' ages were grouped into various categories, with the largest segment, 232 (60.4%) individuals, falling within the 30-45 age range. Educational qualifications varied, with 205 (53.4%) participants holding a bachelor's degree and 85 (4.9%) possessing a doctoral degree. In terms of professional demographics, the sample comprised 249 (64.8%) nurses, 85 (22.1%) doctors, 28 (7.3%) radiology technicians, and 22 (5.8%) other allied health care professionals. A detailed demographic breakdown can be found in Table 1.

**Table 1:** Comparison of the basic and radiological total scores of respondents distributed according to sociodemographic characteristics (N=384) *Significant p-value <0.05; **Significant p-value <0.01; p-value was calculated using Mann Whitney U test or Kruskal Wallis as appropriate Basic knowledge scores were calculated out of a total of 13 points, and Radiological knowledge scores were calculated out of a total of 12 points. IQR: interquartile range

Characteristics	Frequency (percentage)		Total Basic knowledge score, median (IQR)	P-value	Total Radiological knowledge score, median (IQR)	P-value
Gender						
Male		38 (9.9)	11 (9 – 12)	0.245	8 (7 – 9)	0.162
Female		346 (90.1)	11 (10 –12)		8 (6 – 9)	
Did you breastfeed before?						
Yes		259 (74.9)	11 (10 –12)	0.013*	8 (6 – 9)	0.843
No		87 (25.1)	11 (9 –12)		8 (6 – 9)	
Age Group (Years)						
< 30		92 (24.0)	10 (9 –12)	0.011*	7 (6 – 8)	<0.01**
30 – 45		232 (60.4)	11 (10 –12)		8 (6 – 9)	
46 – 60		54 (14.1)	11 (10 –12)		8 (8 – 9)	
> 60		6 (1.5)	12 (11 –12)		10 (8 – 11)	
Profession						
Nurse		249 (64.8)	11 (10 – 12)	<0.01**	7 (6 – 9)	<0.01**
Doctor		85 (22.1)	11 (10 – 12)		8 (7 – 9)	
Radiology technician		28 (7.3)	10 (9 – 11)		8 (6 – 9)	
Other health allied profession		22 (5.8)	11 (8 – 11)		8 (6 – 8)	
Level of education						
Bachelor’s degree		205 (53.4)	11 (9 – 12)	0.013*	7 (6 – 9)	<0.01**
Diploma		89 (23.2)	11 (9 – 12)		8 (6 – 8)	
Board certified		32 (8.3)	12 (11 – 12)		8 (8 – 9)	
Master’s degree		36 (9.4)	11 (10 – 12)		8 (7 – 9)	
Doctoral degree		19 (4.9)	11 (10 – 12)		9 (7 – 9)	
Other		3 (0.8)	12 (7 – 12)		8 (7 – 11)	
Years of experience						
< 5		76 (19.8)	10 (9 – 12)	0.044*	7 (6 – 9)	0.060
5 – 10		104 (27.1)	11 (9 – 12)		7 (6 – 9)	
> 10		204 (53.1)	11 (10 – 12)		8 (7 – 9)	
Employment sector						
Public / Government		306 (79.7)	11 (9 –12)	0.721	8 (6 – 9)	0.996
Private		78 (20.3)	11 (10 –12)		8 (6 – 9)	
Hospital provides CT/ MRI/ nuclear scan?						
Yes		303 (78.9)	11 (9 –12)	0.987	8 (7 – 9)	0.007**
No		81 (21.1)	11 (10–12)		7 (6 – 8)	
Source of information about the topic						
Family, friends, colleagues		69 (18.0)	10 (8 –11)	<0.01**	7 (6 – 8)	0.302
Internet, social media		34 (8.8)	11 (9 – 12)		8 (7 – 9)	
Self-taught		163 (42.4)	11 (10 – 12)		8 (6 – 9)	
Training course		59 (15.4)	12 (10 – 12)		8 (7 – 9)	
Other		59 (15.4)	11 (10 – 12)		8 (6 – 9)	

Overall knowledge score of respondents on breastfeeding practice and radiation imaging 

**Figure 1:**
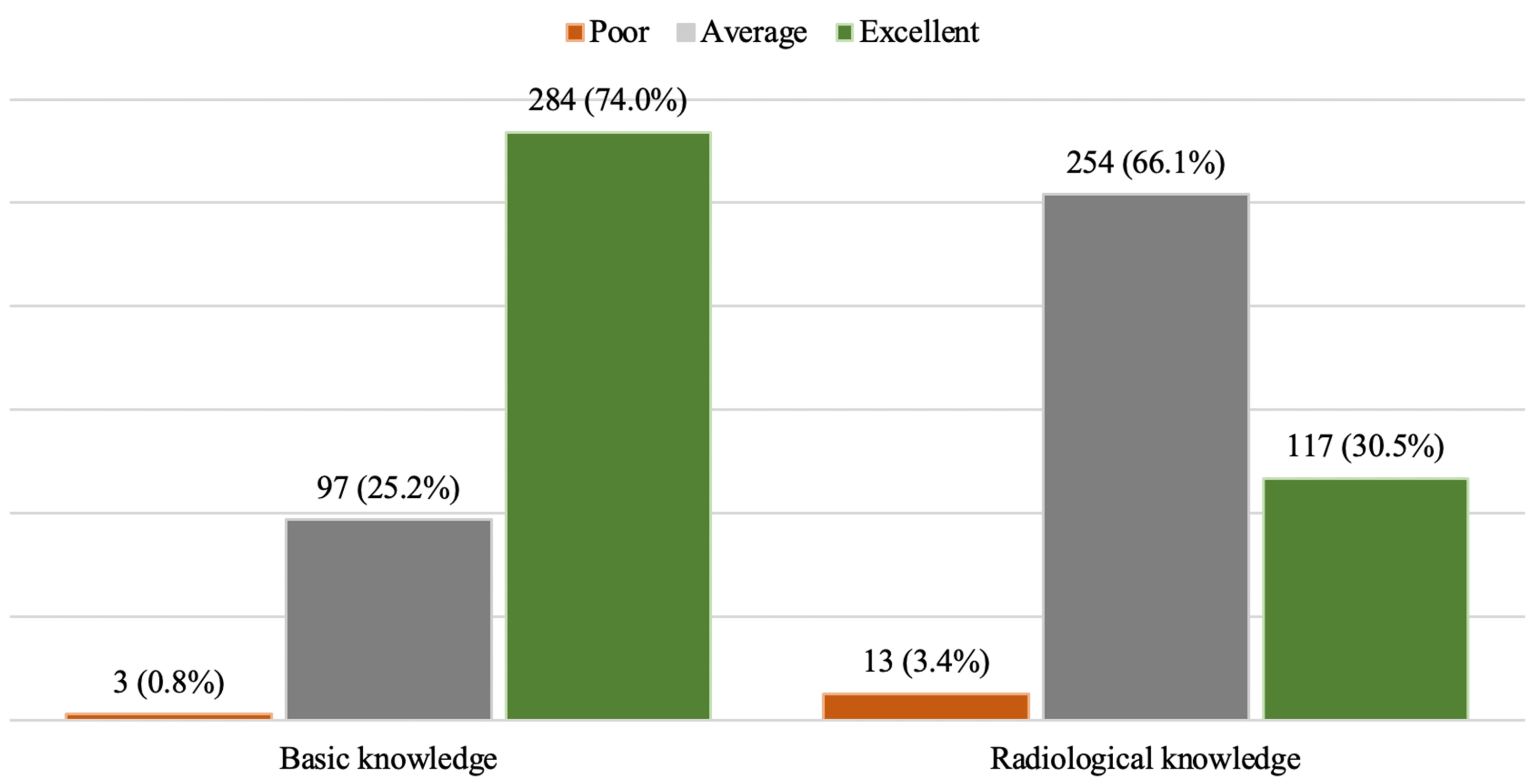
Distribution of basic and radiological knowledge scores (N=384) Data presented as n (%)

A detailed analysis of the respondents' knowledge levels indicated that a substantial majority, specifically 284 (74.0%) individuals, demonstrated an excellent understanding of basic knowledge. In contrast, only 117 (30.5%) respondents achieved an excellent score in radiological knowledge (Figure 1). 

**Figure 2:**
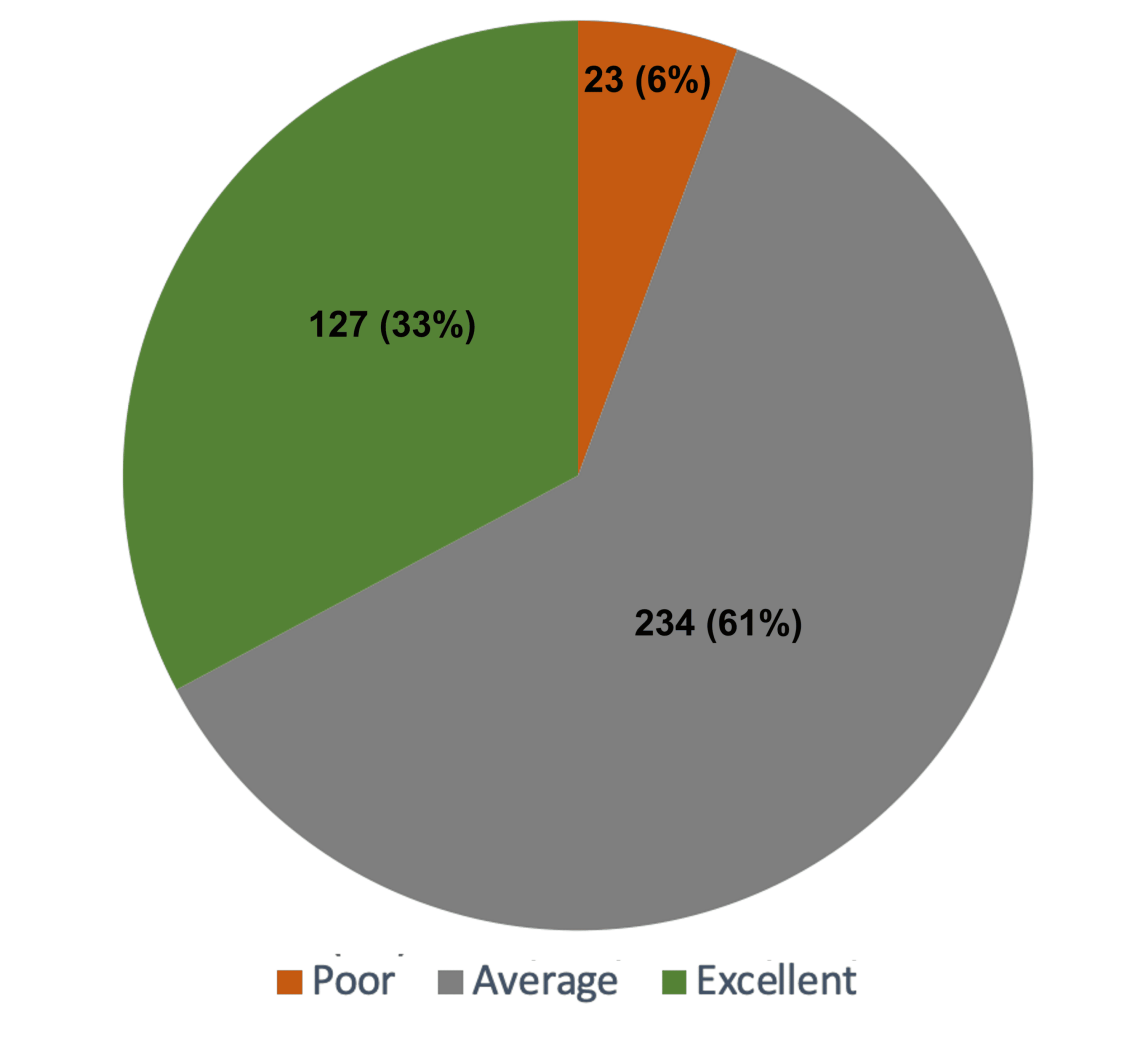
Pie diagram of overall knowledge score

Furthermore, when evaluating the overall combined knowledge score, which encompassed both basic and radiological knowledge, it was found that 234 (61%) respondents reached an average score, while 127 (33%) individuals attained an excellent score (Figure 2). 

Overall, the findings of the study suggest that respondents exhibited a relatively lower level of knowledge regarding radiation imaging compared to their general knowledge of breastfeeding.

Factors associated with knowledge of breastfeeding practice and radiation imaging

The relationship between various factors and the understanding of fundamental and radiation-related knowledge is illustrated in Table 1. No notable differences were observed in the knowledge levels of basic or radiological concepts between female and male respondents. However, a statistically significant difference in median total scores was found between participants under 30 years of age and those over 30 for both basic and radiological knowledge. Among the different educational qualifications, respondents with board certifications achieved the highest scores in basic knowledge, while those holding doctoral degrees had the highest radiological knowledge scores among the various educational levels. 

Individuals with less than five years of experience demonstrated lower scores in fundamental knowledge when compared to their counterparts with over five years of experience. Among various professions, radiology technicians exhibited the lowest scores in basic knowledge, whereas nurses had the lowest scores in radiological knowledge. A notable difference in radiological knowledge was observed between respondents from health centers that offer a range of imaging modalities, such as CT, MRI, and nuclear scans, compared to those from centers lacking such facilities (P<0.01). Respondents who relied on information from family, friends, and colleagues recorded the lowest median scores in basic knowledge, while those who participated in training courses achieved the highest scores. Nonetheless, these information sources did not significantly affect knowledge regarding the use of radiological imaging in breastfeeding women.

Respondents’ replies to the questionnaire

The accurate responses provided by participants to each of the 25 questions concerning Basic Knowledge and Radiological Imaging Knowledge in lactating mothers are detailed in Table 2. 

**Table 2:** Summary of questions, represented as respondent's correct and incorrect answers.

Questions		Correct answer		Incorrect answer	
		Frequency	Percentage	Frequency	Percentage
Questions related to basic knowledge					
1	Breastfeeding is the ideal nutrition for infants	382	99.5	2	0.5
2	Colostrum is the mother’s early milk, which is thick, sticky, and yellowish in color	377	98.2	7	1.8
3	Breast milk provides baby with more protection from allergy compared to formula milk	375	97.7	9	2.3
4	Breastfeeding helps the uterus to contract to its normal size	372	96.9	12	3.1
5	Breastfeeding protects against bone problems like osteoporosis	59	15.4	325	84.6
6	Mothers who practiced breastfeeding have a low risk of getting breast cancer	355	92.4	29	7.6
7	Breastfeeding must be discontinued if mother has breast engorgement	277	72.1	107	27.9
8	Breastfeeding must be discontinued if mother has cracked nipple	258	67.2	126	32.8
9	Breast milk production is influenced by breast size	326	84.9	58	15.1
10	Mothers with inverted nipples can breastfeed their babies	322	83.9	62	16.1
11	Breastfeeding must be discontinued if the infant has jaundice	315	82	69	18
12	Breastfeeding should be continued if the infant has diarrhea	280	72.9	104	27.1
13	Expressed breast milk may be stored for 4 days in the refrigerator, and up to 6 months in a freezer	309	80.5	75	19.5
Questions related to radiological knowledge					
14	The use of radiologic examinations in lactating women has decreased over the past decade	155	40.4	229	59.6
15	It is necessary to counsel the nursing mothers before any radiological procedure	347	90.4	37	9.6
16	Imaging can be divided into modalities, one that uses ionizing radiation (i.e., CT) and the other that do not use ionizing radiation (i.e., MRI and ultrasonography)	332	86.5	52	13.5
17	Breastfeeding should permanently stop after nuclear imaging	232	60.4	152	39.6
18	Iodinated contrasts are not excreted through the mother's milk	255	66.4	129	33.6
19	When radioactive agents are used, mothers should be counseled to pump and store the milk, which can be used later after the agent's radioactivity fades away	254	66.1	130	33.9
20	If the patient is lactating, there is no need to check whether US is more appropriate in imaging than CT	194	50.5	190	49.5
21	When feasible and indicated, MRI is preferred over modalities that use ionizing radiation	314	81.8	70	18.2
22	Ductographic imaging with iodinated contrast agents are not safe to be performed in lactating patients	120	31.2	264	68.8
23	The recommended duration of temporary interrupting breastfeeding should depend on both the physical and biologic half-lives of the different pharmaceutical agents used	337	87.8	47	12.2
24	Before deciding to temporarily stop breastfeeding after intravenous administration of contrast material, the mother should be counseled that the short periods of cessation will not lead to weaning	50	13	334	87
25	Cessation of breastfeeding for a maximum of 12-24 hours can be considered after contrast material administration	321	83.6	63	16.4

**Figure 3:**
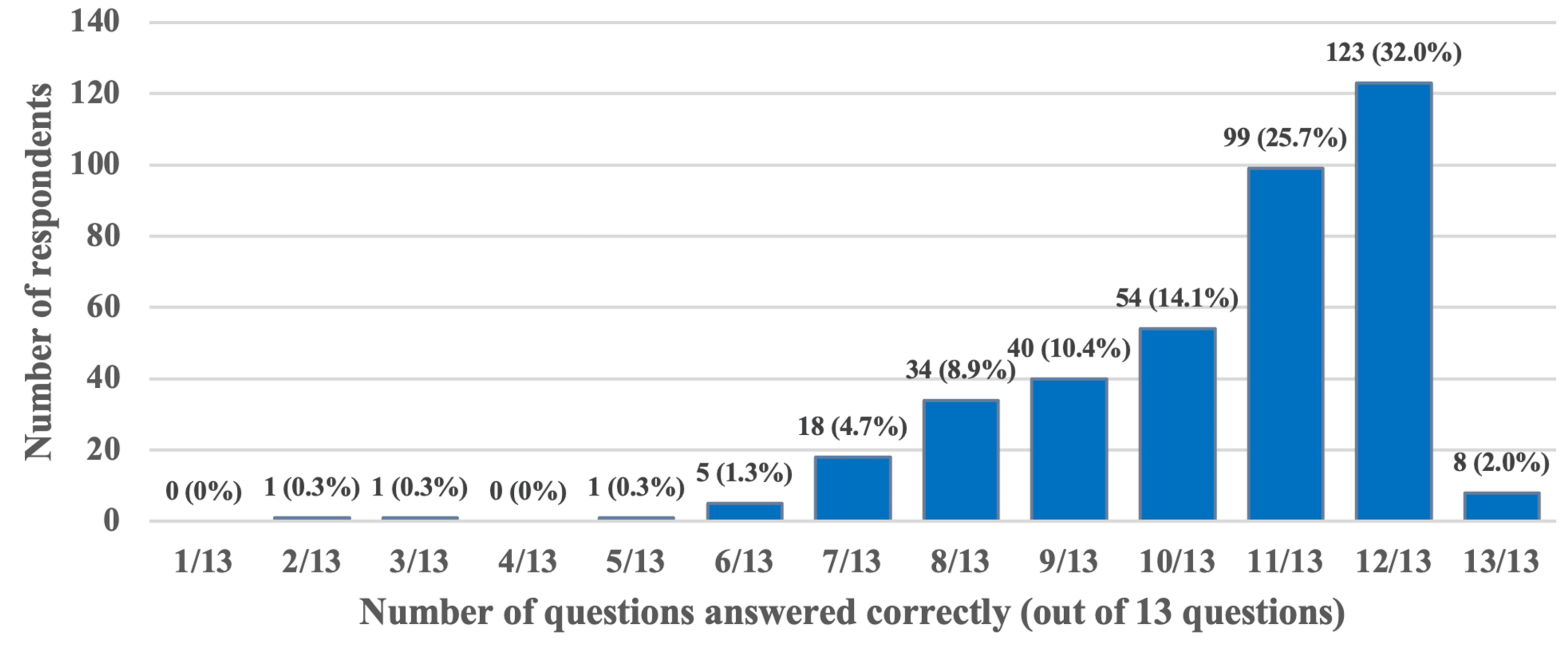
Frequency and percentage of respondent's basic knowledge total scores

Among the questions assessing basic breastfeeding knowledge, the highest number of correct answers was recorded at 123 (32%), with respondents correctly answering 12 out of 13 questions. Only eight (0.2%) individuals managed to answer all 13 questions correctly, as illustrated in Figure 3. One particular question, Question No. 5, was answered correctly by just 59 (15.4%) respondents. 

**Figure 4:**
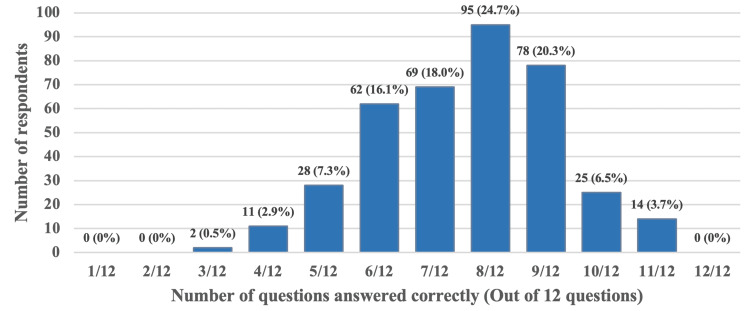
Frequency and percentage of respondent's radiological knowledge total scores

In the second section of the questionnaire, which focused on radiation imaging knowledge, 95 (24.7%) respondents correctly answered a maximum of 8 out of 12 questions, as shown in Figure 4. Three questions received correct responses from fewer than 50% of the participants. These included Question 14 regarding the current status of radiologic examinations in lactating women, with 155 correct responses, Question 22 concerning the safety of ductographic imaging with contrast in lactating patients with 120 (31.2%) correct responses, and Question 24 regarding pre-counseling for mothers about the brief interruption of breastfeeding following imaging with intravenous contrast material with correct replies by only 50 (13%) respondents as indicated in Table 2.

## Discussion

A total of 284 (74%), participants attained an Excellent knowledge score regarding fundamental breastfeeding concepts. In a survey conducted in Nigeria by Ikobah et al., the knowledge score for breastfeeding practices was reported at 85.1% [11], in contrast to our study, which indicated a score of 80.2%. Conversely, a study by Brodribb et al. involving general practitioners in Australia found that 40% of the knowledge-related questions were answered incorrectly [12].

Additionally, our study revealed an average knowledge score of 254 (66.1%) concerning radiological imaging for lactating mothers, with only 117 (30.5%) participants achieving an Excellent score. This discrepancy highlights potential issues regarding the quality of health discussions and counseling provided by healthcare professionals to lactating mothers undergoing radiological procedures in health centers across the Kingdom of Bahrain.

In the current study, both physicians and radiology technicians were included, assuming they possessed a greater understanding of radiological exposure in lactating mothers. However, it was surprised to find that only 50 (13%) of the responses were accurate regarding the question of whether breastfeeding should be temporarily halted following the intravenous administration of contrast material. This lack of knowledge among healthcare professionals may lead to premature recommendations for mothers to stop breastfeeding after radiological procedures involving contrast agents. The American College of Radiology has indicated that it is safe for both the mother and the infant to continue breastfeeding after the administration of contrast agents [8]. They further noted that if a mother has any concerns, she may choose to temporarily discontinue breastfeeding for a period of 12-24 hours, during which she should express and discard milk from both breasts.

The highest levels of knowledge in both basic and radiological aspects were observed among physicians followed by nurses. Our results align with earlier research indicating that medical doctors possess a superior understanding of breastfeeding practices. Okolo and Ogbonna [13] and Chale et al. [14] propose that physicians may play a more significant role in promoting breastfeeding compared to other healthcare professionals. Conversely, a study by Ikobah et al. has indicated that medical doctors may have insufficient knowledge of breastfeeding when compared to nurses [11], which could be attributed to the fact that nurses are primarily responsible for delivering care and education within healthcare settings.

The duration of work experience significantly influenced the foundational knowledge of health professionals. Respondents with less than five years of experience provided a median of 10 correct answers (IQR: 9-12), indicating the lowest knowledge score. In contrast, those with over five years of experience answered an average of 11 questions correctly (IQR: 10-12), reflecting a higher knowledge score, with a statistically significant difference (P = 0.044). This finding contradicts previous research by Chale et al., which found no correlation between years of work experience and basic knowledge of breastfeeding [14]. Additionally, respondents older than 30 years exhibited superior basic and radiological knowledge. However, this observation differs from another study that reported no effect of age on the respondents' satisfactory knowledge levels [11].

In the present study, one of the questions focused on prior breastfeeding practices. The results indicated that healthcare respondents who practiced breastfeeding demonstrated significantly greater basic knowledge compared to those who had not (P = 0.013). Similarly, Brodribb et al.'s study showed that general practitioners with breastfeeding experience possessed enhanced knowledge, experience, and confidence regarding breastfeeding [12]. This correlation is logical, as they acquired insights through personal experience; however, there was no notable difference in radiological knowledge scores between the two groups.

An additional significant factor was the presence of CT and MRI modalities, as well as nuclear scanning, in the healthcare facilities of the respondents. A median of 8 (IQR: 7-9) accurate answers was found to the radiological questions among respondents whose healthcare centers offered CT, MRI, and nuclear scan services, compared to a median of 7 (6-8) correct responses from those without such services (P=0.007). This indicates that the configuration of hospitals and the availability of diverse imaging modalities play a crucial role in improving the knowledge of healthcare professionals in this area.

Many lactating mothers require radiological procedures for various medical assessments, highlighting the urgent need for evidence-based education and effective counseling from healthcare professionals to support their decision to continue breastfeeding. One proposed solution is to implement a standardized, comprehensive breastfeeding education program for healthcare providers. This program should equip professionals with the necessary information to address common breastfeeding scenarios, including the implications of radiation exposure from imaging and contrast agents on both the mother and the infant. It is essential for every healthcare facility that conducts radiological imaging to ensure that its staff is adequately trained to offer the necessary support and education to lactating mothers.

It is important to highlight that there is a lack of published articles in the literature addressing radiological knowledge concerning lactating mothers among healthcare professionals. Consequently, the findings of our study serve as a significant resource for future research. Additionally, this information will aid in formulating effective strategies to enhance the knowledge of healthcare professionals, thereby promoting breastfeeding practices in Bahrain.

One limitation of this study is the predominance of nurses and female participants; this demographic bias may have impacted some of the study's findings, likely due to the fact that female nurses represent the largest segment of the healthcare workforce in the Kingdom of Bahrain.

## Conclusions

The findings of this survey indicate that while health professionals possess an average understanding of the benefits of breastfeeding, their knowledge regarding the implications of radiological imaging for lactating mothers is significantly lacking. Insufficient awareness in this area may lead mothers to discontinue breastfeeding following exposure to radiation imaging. To address these concerns, it is crucial to incorporate information about the effects of radiological imaging exposure into a newly developed breastfeeding education program. This will enhance healthcare professionals' knowledge and ultimately support the promotion of breastfeeding practices.
